# Extensively Drug-Resistant Tuberculosis, Lesotho

**DOI:** 10.3201/eid1406.071654

**Published:** 2008-06

**Authors:** Hind Satti, Kwonjune Seung, Salmaan Keshavjee, Jennifer Furin

**Affiliations:** * Partners In Health, Maseru, Lesotho; † Brigham and Women’s Hospital, Boston, Massachusetts, USA

**Keywords:** Lesotho, mines, drug-resistant TB, migrant labor, South Africa, letter

**To the Editor:** Reports of extensively drug-resistant tuberculosis (XDR TB) in the Republic of South Africa have generated concern in the medical and public health literature (*1*,*2*). Given that XDR TB is spread through the air, it is not surprising that cases have been reported in multiple countries throughout the world (*3*). We report a documented case of XDR TB in Lesotho. This case is closely tied to transnational work in South Africa, thus raising concern about the spread of this disease across highly porous borders and the need for international attention to migrant worker health.

Lesotho is a mountainous nation that is home to 2 million people and completely surrounded by South Africa. It has the third highest rate of HIV in the world; an estimated 24%–30% of the population is infected (*4*). Lesotho also has a high prevalence of TB with an estimated 695 cases per 100,000 population (*5*). Ten percent of patients with smear-positive TB are estimated to have multidrug-resistant TB (*6*). The US Centers for Disease Control and Prevention is undertaking a national reporting registration survey in Lesotho.

In 2007, the Ministry of Health and Social Welfare began working with Partners In Health and the Foundation for Innovative and New Diagnostics, Geneva, Switzerland, to document and treat drug-resistant TB in Lesotho. Hundreds of cases of drug-resistant TB have been reported in the country, and the patient we describe in this letter was reported to have XDR TB.

The patient was a 44-year-old man who had been working in the gold mines in South Africa. He had a history of receiving multiple treatments for TB while he was working in one of the mines. His condition was declared a treatment failure in July 2007. The patient was discharged from medical care in South Africa with no follow-up plan or medical records and was told, per his report, to “return home.” He traveled by road and bus to Lesotho and easily crossed the border. He was originally seen at a public TB clinic in Lesotho but, given his reports of prior TB treatment, his sputum was sent for culture and drug susceptibility testing. He was followed up at his home with a daily visit from a village health worker trained in the management of drug-resistant TB. When XDR TB was confirmed (in vitro resistance to at least isoniazid, rifampin, a fluoroquinolone, and an injectable agent [*7*]), he was admitted to the hospital for drug-resistant TB patients in Lesotho and placed in a negative-pressure, single isolation room.

When the patient sought treatment from our program in October 2007, he exhibited severe wasting and dyspnea. An HIV test result was positive; his CD4 count was 36 cells/μL. First-line drug susceptibility testing (carried out by the Medical Research Council [MRC], Pretoria, South Africa) showed resistance to isoniazid, rifampin, and pyrazinamide. On the basis of these results, on October 26, 2007, he was empirically prescribed a regimen of second-line drugs: capreomycin, para-aminosalicylic acid, cycloserine, ethionamide, and ciprofloxacin. One month later, second-line drug susceptibility testing, sent by the medical team in Lesotho (none was ever sent during his treatment in South Africa) but carried out at MRC, showed additional resistance to amikacin (MIC 1.0 μg/mL), capreomycin (MIC 2.5 μg/mL), and ofloxacin (MIC 1.0 μg/mL) but susceptibility to ethionamide (5.0 μg/mL). The patient’s regimen was changed to kanamycin, moxifloxacin, ethionamide, para-aminosalicylic acid, and cycloserine. Unfortunately, he died of his disease in December 2007. His known contacts are being monitored closely for signs and symptoms of TB. Reports have been made to the mine in which he was working, the Ministry of Health of South Africa, and the Ministry of Health of Lesotho. As of the writing of this letter, the South African Ministry of Health has made no formal response regarding this patient.

The report of this case of XDR TB in Lesotho raises many concerns. First, XDR TB was readily found (along with other forms of drug-resistant TB) in this small country that already has high prevalence of HIV. The potential for spread in the community as well as in hospital settings is substantial. The link to working in South Africa is also a major factor. Given the patient’s prior treatment while employed by a mining company and the literature reporting XDR TB in South Africa (*8*), XDR TB likely developed while he was working in the mines, and he brought the disease back to his home in Lesotho. Because South African mines rely heavily on migrant labor from countries such as Lesotho, Swaziland, and Mozambique, transnational spread of drug-resistant TB, including XDR TB, is quite probable and calls for a concerted international approach and institutional collaboration for management and control of this epidemic. Infection control in the mines needs to be addressed, and international efforts to communicate that migratory populations are at risk for XDR TB need to be a priority in controlling the spread of this disease.
